# Status and Risk of Noncompliance of Adherence to Medications for Metabolic Diseases According to Occupational Characteristics

**DOI:** 10.3390/jcm11123484

**Published:** 2022-06-17

**Authors:** Heeyun Kim, Wanhyung Lee, Jung-Wan Koo

**Affiliations:** 1 Department of Occupational and Environmental Medicine, Central Hospital, Siheung 15079, Korea; heeyunoem@gmail.com; 2 Department of Occupational and Environmental Medicine, Gil Medical Center, Gachon University College of Medicine, Incheon 21565, Korea; wanhyung@gmail.com; 3 Department of Occupational and Environmental Medicine, College of Medicine, Seoul St. Mary’s Hospital, The Catholic University of Korea, Seoul 06591, Korea

**Keywords:** medication adherence, workers, occupational characteristics, metabolic diseases, Korea Health Panel Study

## Abstract

Thus far, little attention has been paid to adherence to medications focusing on the workers and occupational characteristics. This study aimed to assess the status and risk of noncompliance among workers compared to nonworkers, and the association between nonadherence to medication of metabolic diseases and occupational characteristics. Self-reported adherence to medications for hypertension, diabetes, or dyslipidemia and occupational characteristics were evaluated using the Korea Health Panel Study (2008–2018). The status of adherence to medications was evaluated based on working status, with detailed reasons provided for noncompliance. The risk of noncompliance was estimated using the generalized estimating equation, and a subgroup analysis with age-standardized prevalence ratio according to occupational characteristics was also conducted. During the follow-up period, 19,660 (13.9%) person years were noncompliant with medication adherence for 141,807 person years. Workers had a higher prevalence (15.0%) of noncompliance than nonworkers (13.0%). Workers (OR:1.10, 95% CI:1.04–1.14) showed an increased risk of noncompliance compared to nonworkers. Workers who were manual, unpaid family workers, irregular, or dispatched workers showed an increased prevalence of noncompliance. This study found that workers were susceptible to nonadherence to metabolic disease medication. Future research on the role of working conditions in medication adherence would benefit metabolic disease prevention.

## 1. Introduction

Metabolic diseases are the leading cause of cardiovascular diseases and premature deaths worldwide [1,2]. The prevalence and absolute burden of metabolic diseases are rising globally. For example, the rapidly increasing burden of diabetes from 108 million in 1980 to 442 million in 2014 poses a significant threat globally [1] and estimates suggest that 31.1% of adults (1.39 billion) worldwide had hypertension in 2010 [2]. Although lifestyle modifications, such as smoking cessation, exercise encouragement, and a healthy diet, are important factors in the prevention of cardiovascular disease and premature death from metabolic diseases, medication could play a main role [3]. Poor medication adherence to metabolic diseases is closely associated with increased morbidity or mortality and increased unnecessary healthcare expenditures [4]. Compliance with metabolic disease medications is a multifactorial phenomenon. Understanding the status and related factors of adherence to medication for metabolic diseases could be key to preventing cardiovascular disease or death. The World Health Organization defines medication adherence as the degree to which the use of medication by the patient corresponds to the prescribed regimen, which is the starting point of health care [5].

Generally, workers seem to be healthier than the nonworking population, but this is indicated by results from statistics on public health and occupational medicine after the removal of individuals who had health problems in the workplace [6]. Even under the healthy worker effect, a previous study reported that workers showed poorer metabolic disease medication adherence than nonworkers [7]. This might constitute a cornerstone for preventing cardiovascular disease and premature death among workers. To date, there have been few publications on compliance with medication for metabolic diseases, focusing on the working population and occupational characteristics.

This study aimed to identify the status and risk of noncompliance with medication adherence for metabolic diseases including hypertension, diabetes, and dyslipidemia among workers compared with nonworkers and to conduct an analysis of the association between nonadherence to medication and occupational characteristics, including detailed reasons.

## 2. Materials and Methods

### 2.1. Data and Study Participants

**Figure 1:**
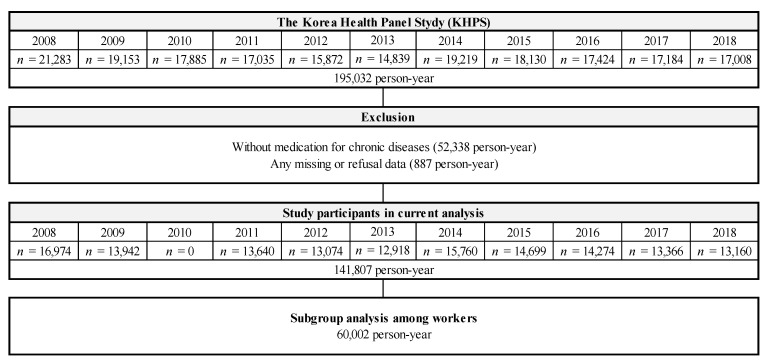
Schematic diagram depicting the study population.

The Korea Health Panel Study (KHPS) is a survey for building panel data that can comprehensively analyze the factors affecting medical use and expenditure, as well as information on medical use behavior and expenditure size. Since 2008, the Korea Institute for Health and Social Affairs and the National Health Insurance Service have conducted the KHPS annually. The KHPS sampled approximately 7000–8000 household members to represent Korean citizens using a two-stage stratified cluster extraction method with probability proportionality from 16 provinces in Korea. Computer-assisted face-to-face interviews were conducted. The KHPS has data on socioeconomic characteristics, health insurance, medical facility visiting information, employment status, metabolic diseases or injury, and health behavior. All data are available upon request at www.khp.re.kr (accessed on 13 April 2022). We used data from the KHPS 2008–2018 (version 1.7.1). We selected 141,807 person years after excluding 52,338 person years who did not have any metabolic disease and 887 person years who had any missing or refusal data from 195,032 person years during the follow-up periods. Figure 1 shows a detailed schematic diagram of the study population.

### 2.2. Main Variables

Medical adherence was assessed using indirect methods, based on self-administered questionnaires [8]. Subjects who answered yes to the question about metabolic diseases diagnosed by physicians and taking medication participated in the current analysis. In the present study, metabolic diseases were defined as patients who needed medication for hypertension, diabetes, or dyslipidemia, according to the Korean Standard Classification of Diseases. Those who had one or more positive answers to the questionnaires about medication adherence for each metabolic diseases were defined as the compliance of medication adherence group, and those who had any negative answers were defined as the medication noncompliance group. Noncompliance groups were asked about specific reasons for nonadherence and these were categorized into the following five groups: relieved symptoms, not being effective, worried about side effects, forgot, and distrust of medication.

### 2.3. Covariables

The socioeconomic variables were age, sex, educational status, household income, and employment status. Education level was classified as middle school, high school, and college or higher. Household income was divided into quintiles. The current working population was grouped as workers.

Health behaviors included smoking, drinking, and exercise. The smoking status was classified according to the current condition: never, past, and current smoker. Severe drinking was defined as averaging more than seven drinks at a time and drinking at least twice a week for men and as averaging more than five drinks at a time and drinking at least twice a week for women. Regular exercise refers to performing two or more moderate-to-intense physical activities per week.

For subgroup analysis of the noncompliance risk pertaining to medication adherence among workers according to occupational characteristics, we used occupational characteristics such as occupational classification, type of employment, job position, contract status, hiring status, and industrial classification. The current study used a modified occupational classification with four categories from the ten categories of the International Standard Classification of Occupations from the International Labor Organization based on a previous study: office, service and sales, agriculture, forestry, and fishing, and manual workers [9]. The office workers included legislators, senior officials, managers, professionals, technicians, associate professionals, and clerical support workers. Service and sales workers included sales and service professionals. The agriculture, forestry, and fishing workers were skilled workers. Finally, manual workers included craft and related trade workers, plant and machine operators, assemblers, and elementary workers. Those in the armed forces were excluded from the study. The types of employment consisted of paid, self-employed, and unpaid family workers. Job position according to work status was included in regular and irregular positions. Contract status was separated into two groups: full-time and part-time. The hiring status consisted of directly hired and dispatched workers. The industrial classification was based on the International Standard Industrial Classification of the United Nations Industry Classification System.

### 2.4. Statistical Analysis

The prevalence of noncompliance pertaining to medication adherence according to baseline characteristics was calculated using the chi-squared test. We used the questionnaires on medication for metabolic diseases and related variables as annual repeated measurement variables from the KHPS during the follow-up period. The odds ratio (OR) and 95% confidence interval (CI) for noncompliance with medication adherence were estimated using a generalized estimating. For sensitivity analysis, the age-standardized prevalence ratio (SPR) and 95% CI on noncompliance of medication adherence to metabolic diseases were calculated according to the occupational characteristics of workers. The prevalence of reasons for noncompliance with medication adherence according to metabolic diseases among workers was also calculated. All analyses were performed using SAS, version 9.4 (SAS Institute, Cary, NC, USA). For all statistical calculations, a two-tailed *p*-value < 0.05 was considered statistically significant.

## 3. Results

The general characteristics of the study participants, according to compliance with medication adherence, are shown in Table 1. During the follow-up period, 19,660 person years (13.9%) were noncompliant with medication adherence. The prevalence of noncompliance with medication adherence was higher in women (14.7%) than in men (12.5%), younger than older, and in subjects with higher education. A high level of household income showed an increased prevalence of noncompliance with medication adherence. The working population had a higher prevalence (15.0%) of noncompliance with medication adherence than nonworkers (13.0%). In terms of healthy behavior, individuals with current smoking, severe drinking, and regular exercise behaviors showed an increased prevalence of noncompliance. According to metabolic diseases, diabetes had a higher prevalence (13.0%) of noncompliance than hypertension (11.9%) and dyslipidemia (12.0%).

Table 2 shows the results from the multivariable generalized estimating equation models adjusted for age in observation year, sex, education, household income level, working status, smoking, drinking, and exercise status. Females (OR:1.37, 95% CI: 1.31–1.44) and workers (OR: 1.10, 95% CI: 1.04–1.14) showed an increased risk of noncompliance with medication adherence compared to males and workers. Current smoking, severe drinking, and regular exercise behaviors were associated with a statistically significant increased risk of noncompliance with medication adherence.

**Figure 2:**
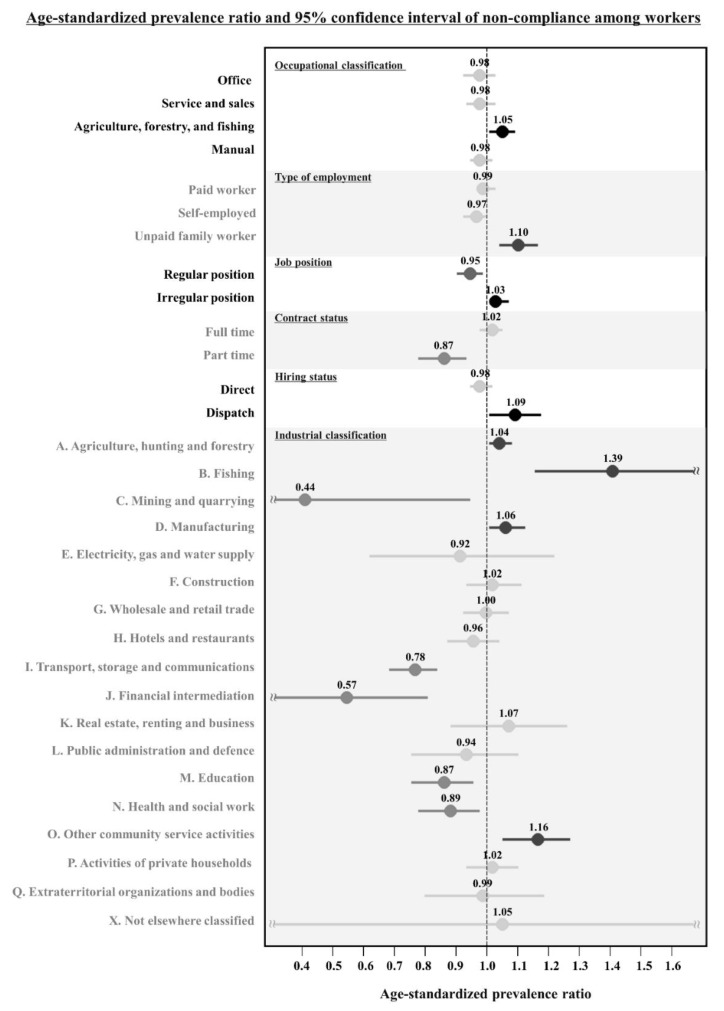
Age-standardized prevalence ratio (SPR) and 95% confidence interval of noncompliance of medication adherence by occupational characteristics among workers (60,002 person years). Black indicates statistically significant positive SPR. Grey indicates statistically significant negative SPR. Light grey indicates non-statistically significant SPR.

Focusing on workers, Figure 2 describes the age-standardized prevalence ratio (SPR) according to occupational characteristics. There was a significant increase in SPR and 95% CI in manual workers (1.05, 1.01–1.11) from occupational classification, unpaid family workers (1.10, 1.04–1.18) from type of employment, irregular position (1.03, 1.02–1.07) from job position, and dispatched workers (1.09, 1.01–1.19) from hiring status, respectively.

**Figure 3:**
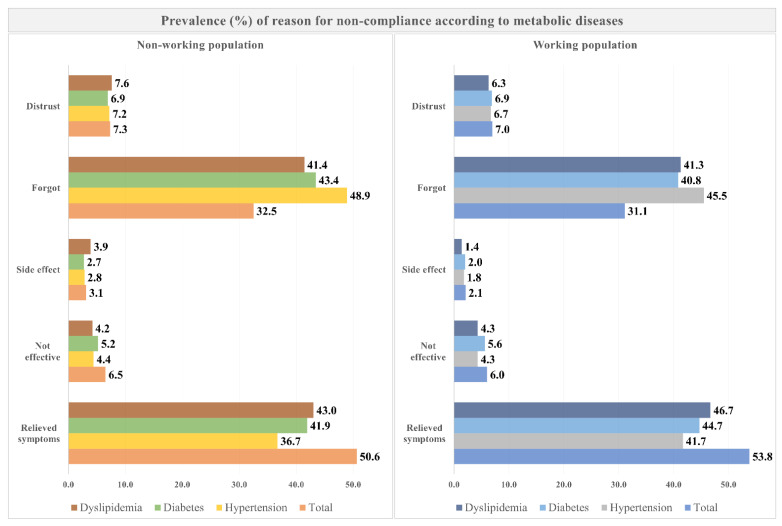
Prevalence of reasons for noncompliance with medication according to metabolic diseases.

Figure 3 shows the prevalence of reasons for noncompliance with medication adherence according to metabolic diseases. Both nonworking and working populations showed the highest prevalence of poor compliance with medication adherence due to relieved symptoms (50.6% and 53.8%, respectively). Most noncompliance with medication adherence for hypertension was due to forgetting medication by both nonworkers and workers.

## 4. Discussion

The current study found that workers had an increased risk of noncompliance with medication adherence for metabolic diseases. According to occupational characteristics, workers who were from the fields of agriculture, forestry, fishing, performed unpaid family work, had irregular positions, were dispatched, and in manufacturing showed an increased risk of poor medication use. Most workers did not take medication for metabolic diseases because of the symptom relief.

There were sex differences in the adherence to medications. This finding is consistent with those of previous studies [10,11]. Sex differences in medication adherence were unclear. A previous study indicated that the differences in medication adherence-related factors such as illness perceptions, causal attribution to balance, causal attribution to risk factors, and personal control were closely linked to the difference in medication adherence between men and women [11]. Although the current analysis could not demonstrate a statistically significant association between socioeconomic status and noncompliance with medication adherence for metabolic diseases, a low level of education and income could be potential risk factors for noncompliance with adherence to medications [12].

Although metabolic diseases could be a key determinant of workers’ working life sustainability or return to work [13,14], very little attention has been paid to their adherence to medications for metabolic diseases. Our results indicate an increased risk of poor adherence to medication for metabolic diseases among workers compared to the nonworking population. The findings will be of interest for the prevention or treatment of metabolic diseases among workers. Further research on the link between occupational characteristics and medication adherence might be helpful in understanding workers’ poor adherence to metabolic diseases. We could identify workers vulnerable to poor medication adherence for metabolic diseases according to occupational characteristics such as occupational classification, type of employment, job position, contract status, hiring status, and industrial classification. According to occupational or industrial classifications, agriculture, forestry, fishing, and manufacturing, workers showed a higher risk of poor adherence to medications for metabolic diseases in our analysis. A vulnerable job status, such as irregular positions or dispatched work, also showed similar results. These workers could be characterized by relatively older age, higher physical demands at the workplace, unsafe job conditions, poor job stability, or lower socioeconomic status than others [9,15,16,17]. These populations are also vulnerable to metabolic diseases or death [18,19]. Older age, higher physical demands at the workplace, unsafe job conditions, poor job stability, and lower socioeconomic status might act as key factors from poor job to poor medication adherence. Further studies regarding the role of occupational characteristics in mediating adherence to metabolic diseases are warranted.

There were few differences in the reasons for inappropriate medication adherence for metabolic diseases between workers and nonworkers according to metabolic diseases. These findings are also useful for educating vulnerable workers on poor medication adherence for the prevention or treatment of metabolic diseases.

To the best of our knowledge, this is the first study in Korea to investigate the association between work status and adherence to medications for metabolic diseases. Furthermore, the current analysis demonstrated an association between occupational characteristics and medication adherence for metabolic diseases.

A major limitation of this study is in the use of a self-questionnaire-based survey. Although self-reported medication adherence is a widely used method, there is a clinical methodology to identify medication adherence with detailed information, such as prescription, medication regimen, type of medication, or dosage. The KPHS did not have various medication adherence-related factors such as ethnic-religious beliefs, symptoms, rural or urban residence, status of medical facilities, or others. Further studies using more detailed information related to medication adherence of metabolic diseases are required. Another limitation was the ecological study design. Ecological studies make these findings less generalizable to individuals.

## 5. Conclusions

This study aimed to assess the association between medication adherence for metabolic diseases and working or occupational characteristics. It found that workers were vulnerable to noncompliance pertaining to the adherence to metabolic disease medication. The second major finding was that there were differences in noncompliance with medication adherence according to occupational characteristics. Appropriate medication adherence is a key factor in the management of metabolic diseases. Thus, further studies regarding the role of work or working conditions would be of benefit in the prevention of metabolic diseases.

## Figures and Tables

**Table 1:** General characteristics of the study participants with metabolic diseases according to medication adherence during the follow-up periods.

Variables	Total, Person Year (% of Column)	Medication Adherence Person Year (% of Row)		*p*-Value
		Compliance	Noncompliance	
No. of participants	141,807 (100.0)	122,147 (86.1)	19,660 (13.9)	
Socioeconomic status				
Sex				<0.0001
Male	55,249 (39.0)	48,315 (87.5)	6934 (12.5)	
Female	86,558 (61.0)	73,832 (85.3)	12,726 (14.7)	
Age (years)				<0.0001
15–20	1518 (1.1)	1097 (72.3)	421 (27.7)	
21–40	5593 (3.9)	4476 (80.0)	1117 (20.0)	
41–60	34,281 (24.2)	28,609 (83.4)	5672 (16.6)	
61–80	86,327 (60.9)	75,561 (87.5)	10,766 (12.5)	
>80	14,088 (9.9)	12,404 (88.1)	1684 (11.9)	
Educational status				<0.0001
Middle School	87,814 (61.9)	75,951 (86.5)	11,863 (13.5)	
High School	35,442 (25.0)	30,483 (86.0)	4959 (14.0)	
College or higher	18,551 (13.1)	15,713 (84.7)	2838 (15.3)	
Household income level				<0.0001
1st quintile	44,185 (31.2)	38,364 (86.8)	5821 (13.2)	
2nd quintile	35,056 (24.7)	30,353 (86.6)	4703 (13.4)	
3rd quintile	24,751 (17.4)	21,235 (86.1)	3416 (13.9)	
4th quintile	20,166 (14.2)	17,127 (84.9)	3039 (15.1)	
5th quintile	17,749 (12.5)	15,068 (84.9)	2681 (15.1)	
Working status				<0.0001
Nonworkers	81,805 (57.7)	71,164 (87.0)	10,641 (13.0)	
Workers	60,002 (42.3)	50,983 (85.0)	9019 (15.0)	
Heathy behaviors				
Smoking status				<0.0001
Never	92,131 (65.0)	78,927 (85.7)	13,204 (14.3)	
Past	32,902 (23.2)	29,041 (88.3)	3861 (11.7)	
Current	16,774 (11.8)	14,179 (84.5)	2595 (15.5)	
Severe drinking				0.0786
No	122,587 (86.4)	105,670 (86.2)	16,917 (13.8)	
Yes	19,220 (13.6)	16,477 (85.7)	2743 (14.3)	
Regular exercise				<0.0001
No	122,897 (86.7)	106,374 (86.6)	16,523 (13.4)	
Yes	18,910 (13.3)	15,773 (83.4)	3317 (16.6)	
Metabolic diseases				<0.0001
Hypertension	34,729 (24.5)	30,605 (88.1)	4124 (11.9)	
Diabetes	19,474 (13.7)	16,949 (87.0)	2525 (13.0)	
Dyslipidemia	16,625 (11.7)	14,630 (88.0)	1995 (12.0)	

**Table 2:** Results of the generalized estimating equation analyzing the risk of noncompliance of medication adherence.

Variables	Noncompliance Risk, Odds Ratio (95% Confidence Interval)
Socioeconomic status	
Sex	
Male	Reference
Female	**1.37 (1.31–1.44)**
Educational status	
Middle School	Reference
High School	0.94 (0.89–1.11)
College or higher	0.97 (0.92–1.02)
Household income level	
1st quintile	Reference
2nd quintile	0.97 (0.92–1.01)
3rd quintile	0.94 (0.90–1.04)
4th quintile	0.98 (0.93–1.03)
5th quintile	0.95 (0.90–1.01)
Working status	
Nonworkers	Reference
Workers	**1.10 (1.07–1.14)**
Heathy behaviors	
Smoking status	
Never	Reference
Past	1.03 (0.97–1.08)
Current	**1.28 (1.20–1.35)**
Severe drinking	
No	Reference
Yes	**1.05 (1.01–1.10)**
Regular exercise	
No	Reference
Yes	**1.10 (1.07–1.14)**

Bold indicates statistical significance. All results were adjusted for age in the observation year, sex, education, household income level, working status, smoking, drinking, and exercise status.

## Data Availability

Data are available at: https://www.khp.re.kr:444/eng/main.do (accessed on 18 May 2022).
